# Inappropriate antidiuretic hormone syndrome presenting as ectopic antidiuretic hormone-secreting gastric adenocarcinoma: a case report

**DOI:** 10.1186/1752-1947-8-185

**Published:** 2014-06-12

**Authors:** Kyungo Hwang, Dae-Hong Jeon, Ha Nee Jang, Eun Jin Bae, Jong Sil Lee, Hyun Seop Cho, Se-Ho Chang, Dong Jun Park

**Affiliations:** 1 Department of Internal Medicine, Gyeongsang National University, College of Medicine, 816 Beongil 15 Jinju-daero, Jinju, South Korea; 2 Department of Pathology, Gyeongsang National University, College of Medicine, 816 Beongil 15 Jinju-daero, Jinju, South Korea; 3 Institute of Health Science, Gyeongsang National University, College of Medicine, 816 Beongil 15 Jinju-daero, Jinju, South Korea

**Keywords:** ADH, Gastric adenocarcinoma, Hyponatremia

## Abstract

**Introduction:**

Although the syndrome of inappropriate antidiuretic hormone has connection with various malignant tumors, there are few reports associated with advanced gastric cancer.

**Case presentation:**

We describe the case of a 63-year-old Korean male with inappropriate antidiuretic hormone syndrome due to an ectopic antidiuretic hormone-producing advanced gastric adenocarcinoma manifested with overt serum hypo-osmolar hyponatremia and high urinary sodium concentrations. His adrenal, thyroidal, and renal functioning were normal, and the hyponatremia improved following removal of the tumor. The cancer cells were immunostained and found to be positive for the antidiuretic hormone. To our knowledge, this is the first report of an antidiuretic hormone-secreting advanced gastric adenocarcinoma associated with the syndrome of inappropriate antidiuretic hormone, showing cancer cells immunostained for the antidiuretic hormone.

**Conclusions:**

Although a strong relationship between gastric cancer and the syndrome of inappropriate antidiuretic hormone remains to be established, we suggest that gastric cancer could be included as a differential diagnosis of cancer that is associated with the syndrome of antidiuretic hormone.

## Introduction

The syndrome of inappropriate antidiuretic hormone (SIADH) is characterized by the excessive release of serum antidiuretic hormone (ADH) relative to serum osmolality. It typically results in excessive water reabsorption in the collecting ducts and hyponatremia. SIADH is frequently found in patients diagnosed with a variety of malignancies, including lung, brain, bladder, duodenum, pancreas, prostate, and head and neck cancer, as well as lymphoma, leukemia, mesothelioma, and thymoma [1,2]. However, there are very few reports of SIADH associated with advanced gastric cancer (AGC) [3-5].

## Case presentation

A 63-year-old Korean male was admitted to our hospital with anorexia, generalized weakness, and melena that began three days prior to admission. The patient had been admitted to the emergency room three months prior due to nausea and vomiting. His serum sodium level was 114mEq/L, osmolality was 250mOsm/kg, and uric acid was 2.9mg/dL. Tests revealed urine osmolality and sodium to be 390mOsm/kg and 57mEq/L, respectively. These laboratory results were consistent with SIADH. Despite a recommendation for further evaluation, the patient refused to be admitted to the hospital. He was subsequently discharged after his symptoms subsided and his sodium levels were restored to 122mEq/L following an infusion of 3% saline.

Upon admission, the patient did not display signs of dehydration or over-hydration. The patient did not have an underlying disease, such as diabetes, hypertension, chronic hepatitis, renal disease, or chronic lung disease, and was not taking any medication. He did not have a contributing family history. His blood pressure was 130/80mmHg, his pulse rate was 78 beats per minute, and his body temperature was 36.7°C. His physical and neurological examinations were normal. Laboratory tests revealed the following: hemoglobin 13.4g/dL, platelets 159×10^3^/mm^3^, white blood cells 6,700/mm^3^ (30% neutrophils, 52% lymphocytes), serum sodium 109mEq/L, potassium 3.6mEq/L, chloride 71.4mEq/L, osmolality 223mOsm/kg, uric acid 2.4mg/dL, alkaline phosphatase (ALP) 110U/L, aspartate transaminase (AST) 23U/L, alanine transaminase (ALT) 29U/L, total protein 7.3g/dL, albumin 3.9g/dL, creatinine 0.9mg/dL, urine sodium 52.4mEq/L, and osmolality 345mOsm/kg. His serum sodium level increased to 121mEq/L following a two-day infusion of 3% saline, and his plasma ADH level (Kit; BÜHLMANN, method: RIA (radio-immunoassay), Schönenbuch, Switzerland) was 11.18pg/mL (0 to 6.7) at that time. Thyroid function tests revealed his thyroid-stimulating hormone (TSH) level to be 1.02μU/mL (0.27 to 4.2), free T4 to be 1.28μU/mL (0.93 to 1.70), and a total T3 of 91.02μU/mL (80 to 200). An adrenocorticotropic hormone (ACTH) stimulation test demonstrated that his basal cortisol level was 22.64μg/dL. Moreover, his cortisol level was 27.89μg/dL at 30 minutes following ACTH administration (250μg), and 33.73μg/dL at 60 minutes after ACTH administration. His prostate-specific antigen (PSA) level was 1.80ng/mL. His serum sodium level partially recovered to 127mEq/L by water restriction prior to surgery.

**Figure 1:**
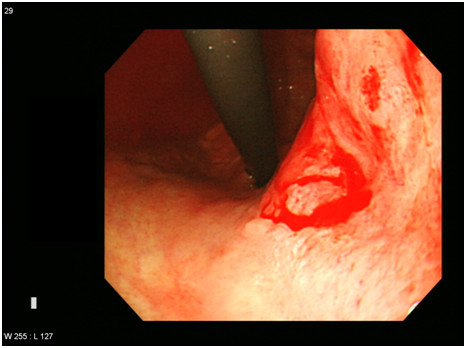
**A gastrofibroscopy shows an ulceroinfiltrative lesion with active oozing bleeding.** The lesion was roughly 2.5 × 2.5cm in size and located at the center of a defective ulcer base of the lesser curvature of the high body.

**Figure 2:**
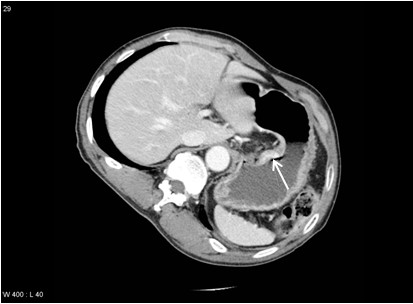
**An axial computational tomography scan of the lesser curvature of the high body in the stomach.** The fat plane is preserved. No lymphadenopathy is seen. Scan shows thickening (arrow) and mucosal enhancement.

**Figure 3:**
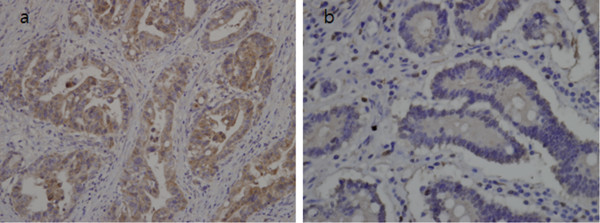
**Immunostainings of cancer and normal gastric cells. (a)** Cancer cells positive for the antidiuretic hormone protein (×200 magnification). **(b)** Normal gastric cells negative for the antidiuretic hormone protein (×200 magnification).

A gastroendoscopy procedure revealed an ulceroinfiltrative lesion on the lesser curvature of the upper high body, with irregular margins and a diameter of 2.5×2.5cm (Figure 1). Bleeding was found to be present at the center of the ulceration. A biopsy revealed a moderately differentiated adenocarcinoma. A computed tomography (CT) scan revealed a thickened wall on the lesser curvature of the high body (Figure 2). A laparoscopic total gastrectomy and Roux-en Y esophago-jejunostomy was performed. Tumor cells were found to have infiltrated the subserosa and had metastasized to a single perigastric lymph node, resulting in stage T3N1M0 cancer. His sodium level and ADH level normalized to 135mEq/L and 5.9pg/mL respectively, three weeks following the surgery (Table 1). Retrospective immunohistochemical staining of the cancer cells were positive for the ADH protein (H-300, Santa Cruz Biotechnology, Santa Cruz, CA, United States) (Figure 3a). No staining was detected on the normal gastric cells (Figure 3b). He died due to cancer progression 1.6 years later after diagnosis despite operation and chemotherapy.

**Table 1:** Serum and urinary laboratory data after admission and post-operation

	**Admission day**	**Admission 3rd day**	**Post OP 1st day**	**Post OP 5th day**	**Post OP 12th day**	**Three weeks after OP**
Serum sodium (mEq/L)	109	121	128	131	134	135
Serum osmolality (mOsm/kg)	223	250	264	276	NA	NA
Urine sodium (mEq/L)	52.4	76.3	73.2	NA	NA	NA
Urine osmolality (mOsm/kg)	325	356	337	NA	NA	NA
Plasma ADH level (pg/mL)	NA	11.18	NA	NA	NA	5.9

ADH, antidiuretic hormone; NA, not available; OP, operation.

## Discussion

Our case report details a patient with SIADH associated with an AGC. This was confirmed by 1) hypo-osmolar hyponatremia, hypouricemia, high urinary sodium concentration and concentrated urine; 2) normal adrenal, thyroid, and renal function; 3) inappropriate secretion of ADH under hyponatremia; 4) positive immunostaining of the cancer cells with an anti-ADH antibody; 5) a full restoration of serum sodium concentration and ADH levels following the removal of the tumor. To the best of our knowledge, this is the first report of AGC associated with SIADH that was histologically confirmed by anti-ADH immunostaining of the tumor cells.

SIADH is often regarded as a diagnosis of exclusions. First, adrenal and thyroid diseases with associated hyponatremia should be excluded. Various medical conditions should be considered in relation to SIADH, including [2]: 1) ectopic secretion of ADH (released from tumor tissue, infections, or conditions with altered intrathoracic pressure, such as with a pneumothorax or status asthmaticus); 2) increased hypothalamic production of ADH-like substances associated with neurological disorders (infections, Guillain-Barre syndrome, and brain tumors); 3) medications (cytotoxic agents, carbamazepine, chlorpropamide, clofibrate, narcotics, and sulfonylurea); and 4) administration of exogenous ADH or oxytocin. Excluding gastric cancer, several conditions were ruled out in the present case by verifying hormone levels and conducting a thorough medical history, physical examination, and laboratory and radiological tests. Importantly, his hyponatremia and elevated serum ADH levels improved following the removal of the tumor, indicating that the hyponatremia was associated with the ADH-secreting cancer cells.

Excessive ADH secretion is often found in tumors with ectopic hormone production. Three clinical conditions are necessary for a tumor with ectopic ADH production [6]. These criteria include SIADH as a clinical symptom of hormone recovery of hyponatremia after removal of the tumor. The hyponatremia present in our patient was due to ADH-secreting cancer cells.

A previous study including a large number of SIADH patients reported that SIADH occurs in 3% of patients with head and neck cancer, 0.7% of patients with non-small-cell lung cancer, and 15% of patients with small-cell lung cancer [1]. The relationship between SIADH and gastric cancer, however, is not well established. Anti-ADH antibodies have not been used to detect ADH in cancer tissue in the three cases of gastric cancer that have been reported since 1990. Here, we demonstrated hyponatremia with ADH-stained gastric cancer cells. Although the rate of gastric cancer is decreasing, it is still the most common type of cancer in Korea. According to the Korean Ministry of Health and Welfare 2010 report, stomach cancer is the leading cause of cancer with an incidence of 15.7%. The use of a gastric endoscopy is difficult to enforce due to the high incidence of stomach cancer. However, the lower incidence of hyponatremia in our case describes a patient who initially presented with symptoms of nausea and vomiting associated with hyponatremia, which lead to a diagnosis of AGC three months later.

## Conclusions

Although a strong relationship between gastric cancer and SIADH remains to be established, we suggest that gastric cancer could be included as a differential diagnosis of cancer that is associated with SIADH.

## Consent

Written informed consent was obtained from the patient’s next-of-kin for publication of this case report and accompanying images. A copy of the written consent is available for review by the Editor-in-Chief of this journal.

## Abbreviations

ACTH: adrenocorticotropic hormone; ADH: antidiuretic hormone; AGC: advanced gastric cancer; CT: computational tomography; PSA: prostate-specific hormone; SIADH: syndrome of inappropriate antidiuretic hormone; TSH: thyroid stimulating hormone.

## Competing interests

The authors declare that they have no competing interests.

## Authors’ contributions

All authors read and approved the final manuscript. HK wrote and edited the report. JDH, JHN, BEJ, CHS, CSH, and PDJ participated in writing and editing the manuscript. LJS helped to describe and interpret our all figures. All authors read and approved the final manuscript.
